# Extracellular ascorbate modulates glutamate dynamics: role of behavioral activation

**DOI:** 10.1186/1471-2202-8-32

**Published:** 2007-05-16

**Authors:** Michael I Sandstrom, George V Rebec

**Affiliations:** 1 Central Michigan University, Psychology Department, Mount Pleasant, MI, USA; 2 Indiana University Bloomington, Department of Psychological & Brain Sciences, Bloomington, IN, USA

## Abstract

**Background:**

A physiological increase in extracellular ascorbate (AA), an antioxidant vitamin found throughout the striatum, elevates extracellular glutamate (GLU). To determine the role of behavioral arousal in this interaction, microdialysis was used to measure striatal GLU efflux in rats tested in either a lights-off or lights-on condition while reverse dialysis either maintained the concentration of AA at 250 μM or increased it to 1000 μM to approximate endogenous changes.

**Results:**

When lights were off, both locomotion and GLU increased regardless of AA dose. In contrast, animals in the lights-on condition were behaviorally inactive, and infusion of 1000, but not 250, μM AA significantly increased extracellular GLU. Interestingly, when ambient light returned to the lights-off group, 1000 μM prolonged the GLU increase relative to the 250 μM group.

**Conclusion:**

Our results not only support evidence that elevated striatal AA increases extracellular GLU but also indicate that this effect depends on behavioral state and the corresponding level of endogenous GLU release.

## Background

Accumulating evidence indicates that ascorbate (AA), an antioxidant vitamin, plays a critical role in modulating striatal function. An increase in extracellular AA, for example, enhances corticostriatal glutamate (GLU) transmission [1,2] as well as the response of striatal neurons to GLU iontophoresis [3,4]. Under naturally occurring conditions, striatal AA release rises and falls, respectively, with increases and decreases in the level of behavioral activity [5,6]. Extracellular GLU [7] and striatal neuron firing rate, which depends on GLU input [8], follow a similar behavior-related pattern. To assess the extent to which the level of behavioral activity influences the ability of AA to modulate extracellular GLU in striatum, we used microdialysis to elevate AA and measure GLU efflux in rats tested under different behavioral conditions. All animals were tested at the same time of day, the onset of the nocturnal phase of the circadian cycle, but AA effects were tested either with the lights on, which suppresses behavioral activity, or lights off, which causes behavioral activation.

## Results

**Figure 1:**
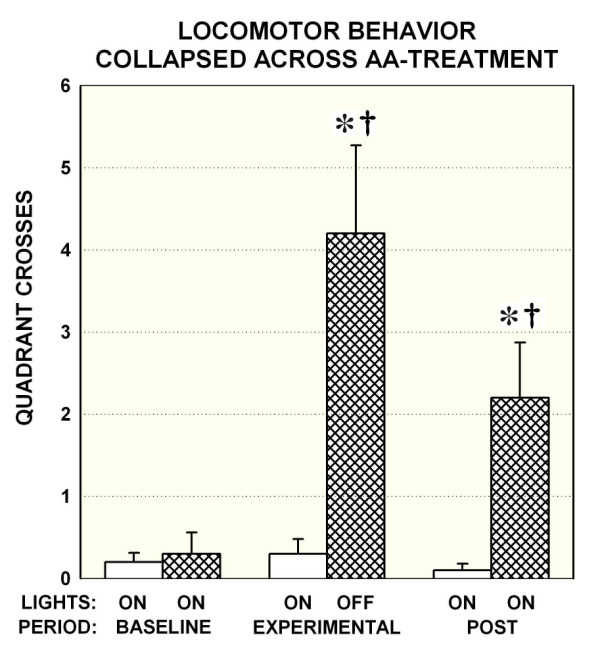
Locomotor responses to light manipulation among the two groups of animals tested: open bars indicate the group experiencing continuous ambient light; hatched bars indicate those experiencing 75-mins of darkness restricted to the experimental period. Mean (± SEM) quadrant crossings according to periods are collapsed across dialysis session in the main graph as AA treatment was found to have no effect on locomotor behavior. Status of lights is indicated by the upper X-axis, and the three consecutive dialysis periods by the lower from left to right. Note the increase in behavior associated with turning off lights during the experimental period, *only among those animals that experienced lights off *(hatched bars) that lingered when lights were turned back on (post period). * p < 0.02 for between-subject comparisons of lights-on versus darkness exposed animals (hatched versus open bars); † p < 0.05 for within-subjects comparisons with initial (lights on) baseline among darkness exposed animals (hatched bars only).

During the baseline period when lights were on, all rats rested quietly and tended to remain in a single corner of the open field. All six rats exposed to continuous light (lights-on) showed minimal to no ambulation throughout the session. In contrast, the six exposed to darkness (lights-off) during the experimental period showed spontaneous locomotion as well as rearing and exploratory sniffing, and this activity persisted, albeit at a lower level, even as ambient light returned in the post-experimental period. Fig. 1 depicts this darkness-induced behavior increase for quadrant crosses. These animals crossed more quadrants than those in the lights-on group during both the experimental [t(11) = -3.53, p < 0.01] and post-experimental [t(11) = -3.02, p = 0.01] periods. While it was expected that returning ambient light (post-experimental) following darkness exposure would result in more immediate behavioral cessation, their decreased locomotion was not significant [t(11) = 2.047, p = 0.065]. Quadrant crosses were unaffected by reverse dialysis of either 250 or 1000 μM AA [p > 0.05 in both cases] ruling out an effect of AA alone on locomotion.

**Figure 2:**
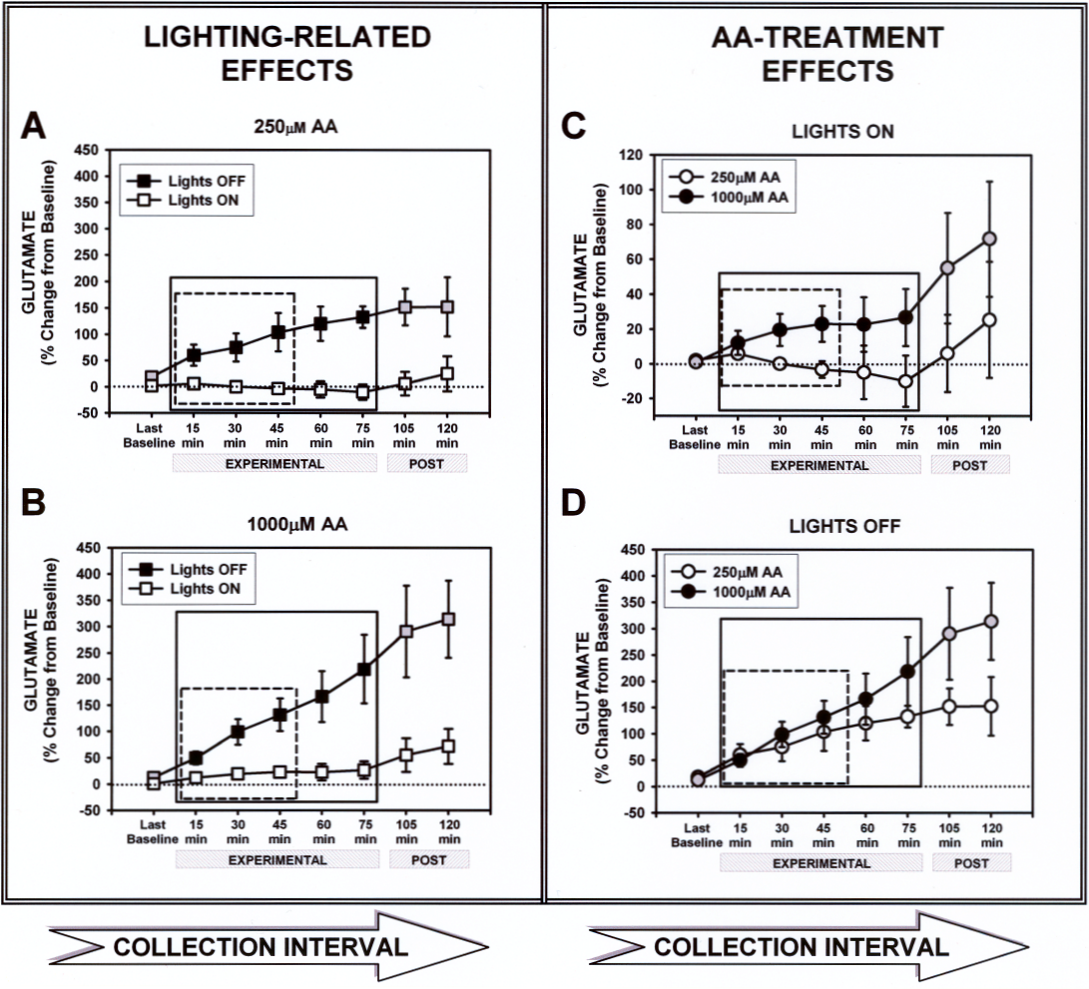
GLU changes are presented as mean percent change from the median of four baseline collections (± SEM) for the dialysates indicated on the abscissa. A combination of 250 μM-AA CSF and "Lights ON" conditions were utilized wherever white or grey-filled icons appear. Black-filled icons indicate when animals were either responding to darkness (A & B), treated with 1000 μM AA (C & D), or both (B & D). The within-subjects factor was AA-treatment, as all animals were treated with both AA levels, 250 μM and 1000 μM AA, in separate dialysis sessions during the experimental period indicated below each graph. Separate graphs depict the GLU level changes occurring in response to lights-out (A & B), or AA treatment (C & D). The boxes within each graph depict time points where separate analyses were performed (last baseline through 45 min dashed, last baseline through 75 min solid). Repeated measures ANOVA analyses within the dashed boxes were significant in A, B, & C. The first 45-min was the only period during which a significant GLU increases in response to AA treatment across collections was exposed, and only during the "LIGHTS ON" experiment (C). Note the large effects of darkness exposure (Experimental period, black-filled icons) on striatal GLU levels depicted in graphs A & B. When the two "Post" collections (black & grey-filled versus white-filled) were included, significant increases in GLU levels occurred across all collections (15–120 min) compared to baseline when animals treated with 1000 μM AA were exposed to darkness (B). However increases in GLU were only significant during the Experimental period (15–75 min) when animals were treated with 250 μM AA (A).

Like quadrant crossings, GLU increased dramatically when lights were off regardless of treatment (Fig. 2A,B). Relative to the lights-on group, which remained behaviorally inactive, lights off triggered a GLU response that increased steadily throughout the experimental period. A repeated-measures ANOVA revealed a significant increase in GLU during either the first 45 min (dashed box) or the entire 75 min (solid box) of the experimental period for both the 250 μM [F(3,30) = 8.95, p < 0.01 and F(5,50) = 6.756, p < 0.01, respectively] and 1000 μM AA infusion [F(3,30)= 9.00, p < 0.01 and F(5,50) = 11.41, p < 0.01, respectively] among dark/active relative to lights-on/inactive animals. Interestingly, when the post-experimental (lights back on) collections were included in the analysis, the GLU increase continued for the dark-exposed animals treated with 1000 μM AA (Fig. 2B) [F(7,70) = 6.50, p < 0.01], but not 250 μM AA (Fig. 2A) [F(7,70) = 2.07, p = 0.06], suggesting that elevated AA can prolong the behavior-related GLU response. In fact, the mean (± SEM) percent GLU increase above baseline for the final post-experimental collection was 314.10 ± 73.10 for the 1000 μM infusion and 152.40 ± 55.90 for the 250 μM group. Thus, not only does behavioral activity increase GLU, but a high concentration of AA can extend this effect.

Analyzing the GLU response by AA treatment (Fig. 2C,D) revealed an effect of 1000 μM during the experimental period but only for the lights-on condition and only during the early phase of this period. Thus, there was a significant effect of AA treatment during the first 45 min (dashed box) [F(3,15) = 3.83, p = 0.03], but not the entire 75 min (solid box) [F(5,25) = 1.81, p = 0.15] of the lights-on condition (Fig. 2C). No AA difference emerged for the lights-off condition (Fig. 2D) at either time [45 min: F(3,15) = 2.73, p = 0.08; 75 min: F(5,25) = 2.29, p = 0.08]. Thus, a darkness-induced behavioral activation appeared to elevate GLU to a level that obscured the AA effect evident in behaviorally inactive animals. Extending our analysis through the post-experimental period revealed no significant AA dose effect for either the lights-off (Fig. 2D) [F(7,35) = 2.06, p = 0.08] or the lights-on group (Fig. 2C) [F(7,35) = 1.15, p = 0.36].

## Discussion

Our results indicate that although an increase in AA can elevate GLU in striatal extracellular fluid, this effect is sensitive to behavioral state. Reverse dialysis of 1000 μM AA increased extracellular GLU during the lights-on condition, when rats rested quietly, but failed to alter the increase in GLU during lights-off when maximal behavioral activity occurred. Thus, the AA-induced potentiation of the magnitude of the GLU response appears limited to behaviorally quiet or less-active conditions when endogenous GLU release is relatively low. There is growing support for increases in GLU release correlating with general behavioral activity. Research on forced-limb use, for example, showed that increased activity of free limbs resulted in increased extracellular GLU in contralateral sensorimotor cortex compared with cortex contralateral to a casted forelimb across extended periods [13]. Regarding discrete periods of behavior, rearing behavior in freely-moving mice has been associated with discrete increases in striatal GLU measured with 13-s resolution [14]. It is possible that the relatively high GLU level during behavioral activity prevented a further AA-induced increase.

When ambient light returned, dark-exposed animals were still active but no longer at their behavioral peak. Under these conditions, prior infusion of 1000, but not 250 μM AA maintained the GLU increase during the post-experimental period. In fact, post-experimental GLU leveled off in the lights-off animals treated with 250 μM. Thus, although the concentration of extracellular GLU may approach a maximal rate of increase during robust behavioral activity, AA appears to enhance this GLU effect by allowing it to continue even as behavior declines toward baseline. Further assessment of the AA-induced change in the behavior-related GLU response is warranted.

The relationship between extracellular AA and GLU appears to depend on the outward transport of AA during GLU uptake [15-17]. Thus, a high level of extracellular AA may prevent GLU uptake by opposing further release of endogenous AA. In line with this prediction, the addition of 500 to 1000 μM AA to striatal extracellular fluid significantly delayed the removal of electrically evoked GLU release [2]. At the neuronal level, moreover, an increase in extracellular AA significantly enhanced the absolute magnitude of the excitation evoked by GLU iontophoresis – albeit without prolonging the response [4]. It appears, therefore, that GLU transmission is regulated, at least in part, by the level of extracellular AA. We show here that in awake animals an increase in extracellular AA can, in fact, elevate striatal GLU release when animals are behaviorally quiet. This effect is less straightforward, however, during behavioral activation.

A likely reason for the complicating effect of behavior is the release of endogenous AA, which, according to AA-GLU heteroexchange [18], would normally accompany the behavior-related increase in GLU transmission. If endogenous extracellular AA is already sufficiently high to interfere with GLU uptake, as would be expected during lights-off when behavior is maximally stimulated, then additional AA will have no further effect. In fact, striatal extracellular AA during behavior can more than double the resting or anesthetized level [18], which ranges roughly between 200 and 400 μM depending on striatal location [19]. Our use of 250 μM in control aCSF should minimize resting AA depletion but allow for endogenous AA release during GLU uptake. Extracellular application of 1000 μM AA blocks the uptake of electrically evoked GLU release from corticostriatal fibers in anesthetized animals [2] and appears to match the extracellular concentration of endogenous AA that can be attained during naturally occurring or drug-evoked periods of behavioral activation [6,20]. As GLU release plateaus and behavior begins to decline during the return of ambient light, endogenous AA release is also likely to plateau, thus allowing for 1000 μM AA to exert a GLU effect.

Interestingly, GLU variability seemed to increase over time (see Fig. 2). A likely source of this variability is the rate of striatal AA perfusion during reverse dialysis. Although animals within a group received the same AA concentration, different AA diffusion rates between probes may have allowed AA to affect GLU at different times during the experimental period. This type of variability may have terminated the AA-induced increase in GLU at 45 min in the lights-on condition (Fig. 2C). It is unlikely that individual differences in locomotion contributed to the variability since animals were consistently inactive (lights-on) or active (lights-off) during the experimental period.

## Conclusion

Our results add to increasing evidence that AA modulates GLU transmission. If, as the heteroexchange model predicts, GLU uptake requires AA release, then the rise in extracellular GLU during behavior may result from a slowing of uptake due to the progressive accumulation of extracellular AA. Other mechanisms also may contribute, including glutamate-cystine exchange since, like cystine, AA is a redox molecule [23]. Although the source of GLU measured in our experiments may include both neuronal and metabolic pools, previous data indicate that the AA-GLU interaction is directly relevant to neuronal release [2]. Because dysfunctional GLU has been implicated in a wide range of clinical conditions from drug addiction to neurodegenerative disease [21,22], further research on an AA-GLU link may have important therapeutic benefits.

## Methods

Male Sprague-Dawley rats (~300 g) were bred from source animals originating from Harlan Industries (Indianapolis, IN). All animals were housed individually under standard laboratory conditions (12-hr light cycle from 07:30 to 19:30) with free access to food and water. Protocols were performed in compliance with the Guide for the Care and Use of Laboratory Animals (1996), and approved by the local Institutional Animal Care and Use Committee. While the animals were under chloropent anesthesia (0.33 ml/100 g ip), a 21-ga stainless steel intracerebral guide cannula (C312G, Plastics One, Roanoke, VA) was inserted unilaterally to the dorsal ridge of anterior striatum: 0.6 mm anterior and 2.8 mm lateral to bregma, and 3.2 mm ventral to skull surface [9]. The guide cannula was secured to the skull with screws and dental cement; an obturator kept the guide debris free. During a two-day recovery period, each rat was habituated to the microdialysis arena (30 × 41.5 cm with sloped sides to allow for ease of movement) for at least an hour.

Intracerebral infusion cannulae (26-ga, C312I, Plastics One, Roanoke, VA) were converted into concentric style microdialysis probes with a 4.0 mm semi-permeable tip of hollow regenerated cellulose fiber (200 μm internal diameter, 18 kD cut off, Spectrum, Houston, TX). All probes were tested prior to each microdialysis session for in-vitro recovery of 75 μM GLU standards at room temperature to yield average recoveries ranging between 8 and 14%. Data from probes with ≤ 5% recovery were not included.

For all microdialysis sessions, artificial cerebrospinal fluid (aCSF: 155.0 mM Na^+^, 2.9 mM K^+^, 1.1 mM Ca^2+^, 0.83 mM Mg^2+^, 132.76 mM Cl^-^, 0.25 mM Na-*l*-AA, and 5.9 mM D-glucose) was pumped through the probe at 2.0 μl/min (see [10]). Dialysates were collected at 15-min intervals in polyethylene autosampler vials and immediately frozen on dry ice. Each session began at 17:00 with insertion of the microdialysis probe followed by a two-hour discard period. The same pattern of dialysis collections were taken from each animal regardless of group (lights-on versus lights off) or treatment administered during the experimental period in each of two dialysis sessions (250 or 1000 μM AA). At 19:00, we collected four 15-min baseline microdialysis samples (baseline period, always 250 μM AA), followed by a 15-min discard period and then five experimental samples (experimental period). During the experimental period, rats received either the baseline aCSF (250 μM AA, control) or aCSF containing 1000 μM AA. Each session concluded with return to control aCSF delivered during two post-experimental collections. Note that control aCSF included a 250 μM concentration of AA to prevent the behavioral impairments caused by depletion of endogenous AA in striatum [11].

Two groups containing six rats each were tested with two different ambient light conditions that differed in whether ambient light was on or off during the experimental period (lights-on and lights-off groups). In the lights-on condition, the lights remained on throughout the microdialysis session. Rats in the lights-off group were always dialyzed in the dark during the experimental period. All rats in each condition underwent two microdialysis sessions, in which control and 1000 μM AA infusions were delivered (during the experimental period) in counterbalanced order, separated by an intervening day of recovery. Control aCSF was always delivered during all other dialysis periods (baseline and post-experimental sessions). Because rats were tested twice, and second dialysis sessions sometimes show decreased transmitter yield compared to the first due to gliosis, a pairwise comparison was performed on median baseline measures corrected for recovery across both sessions for all animals. No significance was found [t(11) = 1.709, p = 0.116], ruling out a session effect. Environmental conditions, moreover, were identical during baseline collections to ensure that all animals began with similar GLU levels.

Behavior (e.g., forward locomotion, grooming, rearing, and sniffing) was observed during collection of each microdialysis sample. Locomotor activity was quantified by dividing the arena floor into four equal quadrants and recording the number of quadrants entered during each observation period; the rat's final quadrant location was recorded to define the starting point for each collection period beginning when a new collection vial was attached. Cumulative quadrant entry counts were then generated for each collection period (baseline, experimental, post-experimental). To observe behavior when lights were off, two red bulbs (7.5 W) were illuminated: one ~60 cm above the dialysis arena, and the other on the adjacent table for recording data.

**Figure 3:**
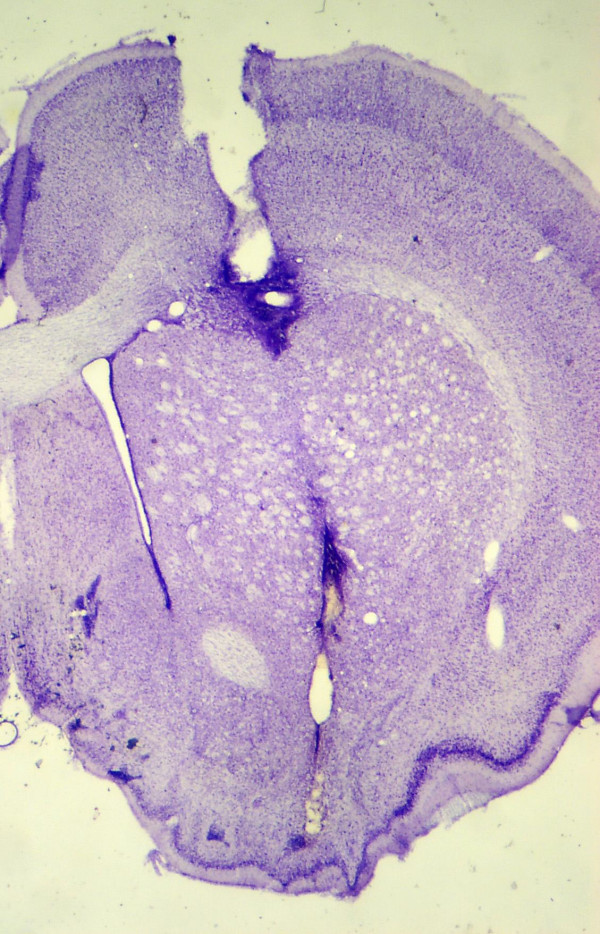
Representative microdialysis probe placement shown in a 40 μm coronal section. Wider cortex damage represents the intracerebral guide cannula termination, while the gliosis seen ventrally shows the typical terminal end of the 4.0 mm semipermeable microdialysis probe tip.

After completion of the second dialysis session, each rat was transcardially perfused with formosaline, and brain slices were mounted on microscope slides for verification of probe placement. Only data from subjects with appropriately placed probe-related gliosis in anterior striatum were analyzed further. A representative section is shown in Fig. 3.

Analysis of dialysate GLU was performed by high pressure liquid chromatography with electrochemical detection (ESA, Bedford, MA) in conjunction with pre-column derivitization (as modified by Yamamoto & Davy)[12]. GLU concentrations were determined by generating a regression curve based on titrated external standards and then correcting for probe recovery by analyzing the in-vitro recovery collections taken before each experiment. GLU concentrations measured in the last baseline, and each experimental and post-experimental sample were expressed as a percent change from the median of the four baseline collections and evaluated by repeated-measures ANOVA assessed against the percent change value obtained from the last baseline collection. Repeated measures ANOVAs were also used to assess the effects of ambient light and AA treatment on locomotion.

## Abbreviations

AA = ascorbate

aCSF = Artifical Cerebral Spinal Fluid

ANOVA = Analysis of Variance

GLU = glutamate

## Authors' contributions

MIS carried out the microdialysis studies, participated in the design of the study, did the initial data analysis, and drafted the manuscript. GVR conceived the study, consulted with MIS on the design, contributed to the data analysis, and worked on the manuscript draft. Both authors read and approved the final manuscript.
